# Retraction: Development and Evaluation of a Simple and Effective Prediction Approach for Identifying Those at High Risk of Dyslipidemia in Rural Adult Residents

**DOI:** 10.1371/journal.pone.0250498

**Published:** 2021-04-16

**Authors:** The PLOS ONE Editors

Following the publication of this article [1], the first author and the corresponding author requested its retraction. The first author and corresponding author stated that the article’s authors do not own the data used in the study and did not obtain permission from the data owners to publish the reported results.

In light of the data ownership issues, the *PLOS ONE* Editors retract the article.

CJW, YQL, LLL, YRG, and RHB agreed with the retraction. LW, LYZ, and MXZ either did not respond directly or could not be reached.
